# An integral forward model of agency experience in thought and action

**DOI:** 10.3389/fpsyg.2025.1524904

**Published:** 2025-01-23

**Authors:** Oliver Lukitsch

**Affiliations:** Faculty of Philosophy and Education, University of Vienna, Vienna, Austria

**Keywords:** action-oriented predictive processing, integral forward modeling, precision weighting, sense of agency, sense of effort

## Abstract

Historically, Frith’s comparator model has been a seminal account of the sense of agency in thought and bodily action. According to this model, only thoughts and actions that are successfully predicted are experienced as agentive, thus providing a unified account of the sense of agency for mind and body. However, this unified model has since been rejected on the grounds that thinking and bodily action impose different constraints on the experience of agency and conscious prediction. While this is widely accepted, the predictive processing model of the sense of agency offers a new perspective that avoids previous arguments against a unified comparator model and paves the way for its reintroduction.

## 1 Introduction

When I move my arm, I feel that I am the author of my bodily movement. When I think I will take the tram rather than the bus, I feel I am the author of my thoughts—or that I am the one generating those thoughts. Can these two experiences be explained by a singular mechanism? A unified account of the sense of agency (SoA, for short) would establish the theory that a single mechanism can explain SoA in bodily movement and thinking. This paper aims to show that, against the odds, such a unified comparator account of SoA is possible. The mechanisms underlying SoA for bodily movement and thinking have traditionally been understood as distinct, and the burden of proof is on the proponent of the unified account, as time and time again, arguments against a unified comparator account of SoA have been put forth. We shall revisit them one by one in this article. In summary, they hold that there is a categorical difference between predicting our actions and the course of our thoughts. That is, thought and action are too different to be governed by a single predictive mechanism. First, however, let us revisit the unified comparator model and the rationale for its original introduction.

### 1.1 Historical context: the unified comparator account of SoA revisited

It was Frith (1992) work on schizophrenia that kickstarted the exploration of SoA. Frith’s idea was that a single predictive mechanism, called “forward modeling,” can explain a wide range of both bodily and mental symptoms in schizophrenia, specifically so-called “passivity experiences.” These experiences are characterized by the affected person feeling that their thoughts or bodily movements are under the control of an external force or person rather than being self-generated. A unified model, therefore, kills two birds with one stone, claiming that such symptoms arise due to a shared lack of SoA – and it explains the lack of SoA in terms of a single underlying mechanism.

Frith identified the cause of all experiences of passivity as the disruption of a predictive mechanism that matches the predicted effects of action and thought with their actual effects. However, this idea quickly lost its appeal because, on closer examination, thought and action are too dissimilar to be coherently dependent on a single predictive mechanism. In short, there seems to be a categorical difference between the way we anticipate our actions and our thoughts. To take just one example, consciously predicting the sensory effects of an action is clearly different from consciously perceiving the effects of the action (after the fact). But to predict that I will think *p* is arguably to think *p*.

More recent developments in cognitive science point in a different direction, however. They provide a more nuanced understanding of bodily SoA – and I will show that such a more nuanced characterization of SoA paves the way for a revised unified comparator model. This revised comparator account, does not suffer from the conceptual problems that a unified model is said to be unable to overcome. In particular, I suggest that the framework of “integral forward modeling” (Pickering and Clark, 2014; Clark, 2016) can serve as a conceptual basis for a revised unified framework.

This paper is structured in four parts. First, I will show that several arguments against a unified account are based on one idea: that SoA is a quasi-perceptual experience, or that it has a mind-to-world direction of fit. Second, I will present more recent comparator models based on the principles of predictive processing and *integral forward modeling*. These models both characterize and explain SoA in terms of its temporally extensive structure. According to such accounts, SoA is not a quasi-perceptual experience. Instead, they opt for a telic model that grounds SoA in motor commands rather than descriptive states. Third, I will turn to the arguments against a unified account and revisit them one by one through the lens of a predictive processing account: the goal is to resolve the conceptual problems of a unified comparator model in doing so. And forth, I will touch upon the prospects of a unified predictive processing account in the light of recent evidence in the study of SoA.

I am, however, not alone in arguing that a predictive processing framework can explain SoA with reference to the same machinery in thought and bodily movement. For example, while (Allen and Friston, 2018; Leptourgos and Corlett, 2020) assume that predictive processing accounts can provide such a unified comparator model of SoA, they do not answer to the criticism that challenges and abandons the unified model. The key contribution of this paper is to address this criticism and dissolve it.

## 2 The sense of agency and the comparator model 30 years on

The understanding of SoA has evolved significantly since its introduction. In short, it initially referred to mind and body while referring only to bodily movement or action today. Today, Christopher Frith (1992) theory of disrupted self-awareness in schizophrenia is seen as an outdated, but seminal proposal for a holistic model. Frith argued that people with schizophrenia lack the ability to represent their own mental states (i.e., meta-representation) and are therefore unable to track their agency.

He explains this phenomenon by the disruption of a “comparator” mechanism that compares or matches predictions of action effects with outgoing motor commands. The primary purpose of this mechanism, however, is not for tracking a subject’s agency. Rather, the comparator primarily serves the purpose of (motor) control, allowing motor commands to be adjusted *before* an action yields its effects. Such a mechanism must not be confused with comparing action effects and predicted action effects *after the fact*, i.e., after the occurrence of action.

Frith went on to claim that a comparator mechanism and its disruption are the cause of disrupted SoA in thinking and bodily action. Such a unified account is an appealing, parsimonious explanation of a wide variety of schizophrenic symptoms. That is, a single disruption explains the variety of aberrant experiences in the schizophrenic spectrum, usually summarized as “passivity experiences.” Some passivity experiences coincide with bodily action such as delusions of control. Others occur in thought, such as the symptom of thought insertion and or thought withdrawal.

### 2.1 Different conditions of prediction in motor control and thought

Frith held that the comparator mechanism serves the phylogenetically primary purpose of *motor* control and the fine-tuning of movement. More recent versions of the comparator model adopt this original view: The ecological selective pressure for the evolution of the comparator mechanism is the control of bodily movement (Wolpert and Kawato, 1998; Gallagher, 2004b). The ability to predict the effects of movement allows a cognitive system to adjust the course of the movement (even before it takes effect). It also allows the cognitive system to confirm that a movement has been carried out as intended.

This is where we arrive at the focal subject of this article. The comparator’s primary purpose or biological function does not plausibly carry over to thought. To put it differently, one might ask: Is there a need for a comparator in thoughts at all? Do the effects of thought need to be predicted to begin with, and if so, in the same way as bodily movements? Gallagher (2004b, 2005) denies this, arguing that the ecological pressure for a comparator mechanism does not arise in thought. First, unlike my bodily movements, my thoughts are my thoughts by default. Therefore, no evolutionary pressure should have led to the emergence of a mechanism that verifies the thinking subject’s agency. And second, neither will a comparator be needed to adjust the course of thoughts like it would adjust and adapt the course of movement against the backdrop of external contingencies.

Although Campbell (1999) and Gallagher (2005) do not say so directly, their analyses imply that, unlike thoughts, bodily movements are influenced by external contingencies or the environment. Because bodily movements interact with the external world, motor control must account for and adapt to potential uncertainties. On the other hand, thought and mental action are generally less affected by external conditions than are bodily movements. The emphasis here is on *less.* Our thoughts do not always unfold as intended, and contingencies can interfere with a predicted line of thought. Thus, Campbell (1999) has suggested thinking may require a mechanism that keeps our thoughts “on track” by continually evaluating whether they are going in the right direction. In other words, the biological function of the comparator may be to keep thoughts on a coherent semantic path.

However, this does not save the day for the holistic model, according to Gallagher (2004b, 2005): if a comparator mechanism exists to keep our thoughts on track, then it cannot be a conscious process. For we do not consciously compare predictions of what we are thinking and what we intend to think. The worry is that such a conscious mechanism would arguably produce a “double awareness” of thought content—where the experiential difference between predicted thought and thought remains unaccounted for. Campbell (1999) thus suggested that since there is no such double awareness in human experience, the comparator must operate subpersonally and that it does not yield any conscious experience of prediction. The problem with this idea is, however, that a comparator mechanism operates subpersonally might be considered redundant, given that conscious monitoring of thought is sufficient to keep it on a coherent semantic track (Gallagher, 2004b, 2005).

For the present discussion, the crucial implication is that thinking and bodily action impose different constraints on a comparator model of SoA. The difference arises because prediction plays a different role in thinking than it does in bodily action. For example, we can be surprised by how the course of our actions unfolds. Yet, our thoughts rarely surprise us like a physical action being interrupted by an external event (although they can under abnormal psychological conditions). Moreover, the different conceptual constraints that bodily movement and thought place on prediction and SoA become even more evident in the more recent version of the comparator model as SoA is increasingly understood as a perceptual awareness of prediction. In a nutshell, if SoA is characterized as a perceptual awareness than it cannot ensue in thought—as perceptual experience is limited to the experience of external events, rather than thoughts. The ensuing section will delve into this in more detail.

### 2.2 The evolution of the comparator: SoA as perceptual awareness

Frith’s original comparator model (and theory of schizophrenia) has undergone significant revisions since its introduction (Blakemore et al., 2000, 2002; Frith et al., 2000; Jeannerod, 2009). The original comparator model held that the comparison between prediction and predicted outcome happens prior to the occurrence of action effects by matching motor intentions with predicted action-consequences. Moreover, it understood prediction as an underlying mechanism of agency experiences (Frith, 1992).

In contrast, more recent findings by Tsakiris and Haggard (2003) and Synofzik et al. (2009) suggest that SoA results from the comparison of *predictions* of action effects and *actual* action effects.

Most importantly, more recent comparator models of SoA *characterize* SoA (i.e., describe the *explanandum*) as an awareness of prediction (Blakemore et al., 2000, 2002; Frith et al., 2000; Jeannerod, 2009). Prediction and forward modeling is thus not just a subpersonal mechanism—but a *eo ipso* conscious phenomenon giving rise to SoA. In other words, SoA has been described as awareness of predicted (proprio- and exteroceptive) consequences of action (i.e., awareness of one’s predicted limb position relative to the environment). The resulting SoA could then be diminished or disrupted if these predictions fail. For example, I want to reach for my cup of coffee, but my arm will not comply. Therefore, I will lack SoA for the intended movement because I was aware of a prediction that did not succeed. In other words, most recent version of the comparator model (Jeannerod, 2009; Synofzik et al., 2009; Haggard, 2017) describe the experience of agency as the perception of *bodily action* (as a result of the works of the comparator).

Yet, the literature in cognitive neuroscience tends to be vague, often relying on intentional binding (Haggard et al., 2002) as an intersubjective indicator of SoA, while undercharacterizing the experience of agency. From a philosophy of mind perspective, however, and in line with the comparator model, Tim Bayne in particular has recognized and addressed this often-neglected characterization that SoA as a perceptual awareness. He emphasizes that the first-hand *qualitative character* of agency ought to be described as *perceptual*, or yielding a “perceptual model of agentive experience” (Bayne, 2011). By matching action-predictions and effects, the comparator gives rise to such first-hand perceptual awareness of authorship^1^.

For the article at hand, however, it is crucial to understand that a perceptual approach to SoA, as portrayed above, exacerbates the odds for a unified model. Typically, perceptual systems are oriented towards the external world, rather than internal states such as thoughts. Because our bodily actions unfold in the external environment, it is plausible that a perceptual system is involved in verifying the authorship of our bodily movements—all the more so as we perceptually monitor the external world as we act upon it in the first place. To illustrate, the movement of our limbs can be caused by ourselves, but it can also be caused by an external force, for instance, when someone moves our limbs for us. Our thoughts, on the other hand, cannot be caused by external forces in the same direct way. Most importantly, while we can monitor our thoughts, we do not monitor them *perceptually*. Therefore, by delineating SoA in bodily action as a perceptual experience, the phenomenology and its explanation are limited to monitoring events in the external world.

### 2.3 SoA as non-perceptual awareness

The perceptual notion characterized in the previous section, however, is not an unrivalled portrayal of SoA in the literature. In the framework of the comparator model, the experience of agency is sometimes described as a *non-perceptual, non-observational* awareness (Gallagher, 2004a, 2005, 2007; Gallagher and Zahavi, 2012; cf. Shoemaker, 1968) or as pre-reflective experience or “feeling” of agency (Synofzik et al., 2008; Grünbaum, 2015; Bermúdez, 2024; Malik et al., 2022).

The key difference between the perceptual concept of agency and the non-observational concept is that they involve different directions-of-fit. Bayne illustrates this difference, conceptualizing it in terms of thetic and telic notions of sense *of* agency respectively:

“The thetic theorist reads this “of” intentionally: The phenomenology of agency involves experiences that are intentionally directed toward agency. By contrast, the telic theorist reads this “of” possessively: The phenomenology of agency is a matter of one’s actions (or tryings) themselves having experiential character.” (Bayne, 2011, p. 363).

This difference is of essence for any explanatory model of SoA. If we understand SoA as perceptual experience, then we understand it as a *descriptive* (or at least non-conceptually representational) experience. If we understand SoA as resulting from the efferent machinery of action-production, then we understand it as *directive*.

A non-observational, pre-reflective concept of agency is intuitively appealing. For it dodges the counter-intuitive notion that the experience of ourselves as causing an action is not just an observation of an external event but establishes a different type and quality of experience. In other words, it rejects the idea that SoA is nothing but a mode of watching oneself do things. Instead, access to one’s actions (and the fact that they are self-produced) is more closely grounded in the machinery of action *production* (rather than action monitoring). This notion is conceptually rooted in Anscombe’s idea of *practical knowledge*, i.e., the idea that we do not know that we are the authors of our actions by observation (Anscombe, 1976).

Furthermore, a non-observational concept of SoA is appealing as it supports the distinction between perceptual judgments of agency and the first-hand experience or feeling of agency. After all, we *can* perceptually judge that we are the authors of our bodily movements, but *before* we do so, our movements feel self-produced (Malik et al., 2022). A non-observational notion can account for this difference.

Whichever concept of SoA one chooses, by it observational or non-observational, they will constrain a (reductive) explanation of SoA in different ways (due to the different causal roles SoA plays given their different conceptualizations). Yet, the debate over the scope and details of the comparator model of SoA tended to ignore this conceptual difference. Notwithstanding, recent evidence suggests that the characterization and phenomenology of SoA has been oversimplified and that the picture is more nuanced.

## 3 Deconstructing SoA

Rather than siding with a telic or thetic approach to characterizing SoA, more recent findings suggest that the phenomenology of SoA is both multifaceted and temporally extensive. As stated in the previous section, it is said to involve *both* the perceptual awareness of action, but also non-observational (or telic) aspects. Accordingly, it is the interplay of these components over time that give rise to the fully-fledged experience of agency.

The orthodox comparator account simplifies SoA to the post-hoc perceptual awareness of *successful* action-prediction. As opposed to this, recent discussion of SoA (in the context of the comparator model) claim that the orthodox understanding of SoA is exclusively based on the idea of *successful* prediction, but that successful prediction *is not sufficient* for the experience of agency (Gerrans, 2014; Zaadnoordijk et al., 2019). Rather, the experience of agency also includes experience of *effort*, oftentimes against the backdrop of uncertainty and precariousness (Gerrans, 2014; Lukitsch, 2020; Bermúdez, 2024; Bermúdez and Massin, 2023). Taking this idea further, Lukitsch (2020) argued that SoA is a composite experience necessarily containing a “sense of effort” and a “sense of efficacy.” On this account, the sense of effort is defined as the “experience of resistance and difficulty that increases the more an action deviates from its goal” (ibid. p. 959). The sense of efficacy, on the other hands, merely confirms that actions are unfolding as predicted, underlining the effectiveness of one’s actions (rather than authorship). The orthodox model of SoA then reduces the sense of *agency* (and confuses it) with a much narrower sense of *efficacy*.

Note that such a composite notion of SoA consisting of effort and efficacy seems inconsistent at face value. It defines SoA in terms of an awareness of both successful and unsuccessful prediction. Still, this inconsistency is only apparent, as SoA is defined as temporally extensive, suggesting a structured interplay of both successful and unsuccessful prediction. In line with this temporal perspective, some authors have emphasized the significance of temporal nature of the experience of agency.

### 3.1 The temporal structure of SoA

In their recent work, Bermúdez and Massin suggested that the sense of effort may be linked to (the ongoing process of) minimizing prediction error (2023). This contrasts with Pacherie (2008) characterization, which equates the sense of effort with momentary prediction error. Thus, while Pacherie views effort as a static outcome resulting from prediction discrepancies, Bermúdez and Massin imply a dynamic perspective that emphasizes the continuous effort to align predictions with outcomes.

In a similar way predictive processing accounts conceptualize (bodily) SoA as the experience of gradually and successfully overcoming prediction errors over time (Gerrans, 2014). SoA is thus realized through the temporal structure of prediction error minimization (Lukitsch, 2020). A notable difference to (Bermúdez and Massin, 2023) is that the predictive processing account considers effort and the awareness of successful action-prediction as phenomenologically intertwined aspects of agency experience. For example, an unfolding action will be experienced as agentive if it is predicted successfully, but only against the backdrop of overcoming prediction-error.

Hence, a predictive processing account of SoA presupposes an inherent antagonism of successful prediction and prediction-error. SoA, then, arises as a result of the temporally extended process of error-cancelation. To illustrate, when navigating over an unsteady, rocky surface, our motor system is continually confronted with new contingencies it must account for. It is through the very process of integrating such contingencies in ongoing bodily action that we come to experience SoA (Gerrans, 2014; Clark, 2016; Lukitsch, 2020).

### 3.2 Leeway for a unified account

To close the circle: Criticism and rejection of the holistic model of SoA was based primarily on the notion that SoA is a perceptual or thetic experience. This criticism, however, neglects the efferent aspects of the experience of action, suggesting that the machinery of action *production* does not contribute to the SoA in any significant way. In contrast, and as discussed above, more recent definitions of SoA claim that it is a multifaceted experience also involving *efferent* commands and the awareness thereof. This paves the way for a telic concept of agency in both action and thought. For while a thetic perceptual conception of SoA fails to capture the phenomenology of agency in thought, no compelling argument has been put forward against a telic approach to SoA.

Most importantly, at least for the present article, a unified account of SoA can accommodate both perceptual and efferent aspects, thus providing a more comprehensive understanding of agency. To effectively integrate these multifaceted components of SoA, it is parsimonious to adopt a theoretical framework that can encompass both the perceptual and efferent dimensions. Predictive processing turns out to be a promising candidate for such an integrated account. It provides the necessary framework to explain the temporally extended and dynamic nature of agency. Furthermore, it also integrates the perceptual and efferent aspects of SoA, allowing for a more nuanced understanding of agency, thus addressing the conceptual tensions discussed in the previous section.

## 4 Predictive processing

At the heart of this article lies the notion of a unified comparator account in the framework of predictive processing (Friston, 2012; Clark, 2013, 2016; Hohwy, 2013). In short, predictive processing explains the multifaceted phenomenology of agency as the temporally extensive process of prediction error cancellation (Gerrans, 2014; Lukitsch, 2020). To illustrate this, consider driving a car in heavy rain or snow. The slippery roads, reduced visibility and unpredictable behavior of other drivers require you to be constantly alert, adjusting your speed and direction, thus increasing your SoA. On the other hand, if you drive on a clear, straight motorway on a sunny day with minimal traffic, your actions become almost automatic and your SoA may feel less pronounced. Under difficult conditions, you are more likely to encounter increased prediction error. Yet, at the same time, you are also more prone to decreasing the error signal, compensating for possible missteps. This continuous process of high weighting of prediction errors and their continuous cancellation results in SoA, according to predictive processing. For the discussion at hand, however, it is crucial to understand how predictive processing integrates both telic (goal-directed) and thetic (perceptual) elements of SoA in its predictive machinery. To do so, I will examine the difference between the modalities of forward modeling in both predictive processing and the traditional comparator model.

### 4.1 Integral forward modeling: a departure from the orthodox comparator model

Predictive processing and the comparator model exhibit some conceptual similarities. Yet, there exists a principled difference between them (e.g., Bayne and Pacherie, 2007; Frith et al., 2000). The orthodox comparator model is described as a specialized module dedicated to anticipating the sensory effects of bodily actions. Pickering and Clark (2014) refer to this version of the comparator model as “auxiliary forward modeling” (AFM). AFM accounts of motor control claim that forward modeling happens in isolation from other mechanisms, such as those involved in action-production, such as the inverse model that is used to compute the motor command. In short, AFM understand the comparator mechanism as a compartmentalized special-purpose module for prediction. It holds that a motor command will yield an efference copy of that command. The latter is then used to create a forward model, predicting the sensory consequences of the motor command. Crucially, the traditional comparator model is full-fledged instance of AFM.

In contrast, predictive processing accounts (Adams et al., 2013; Pickering and Clark, 2014; Clark, 2016) hold that forward modeling is not a compartmentalized support module for assisting motor control. Instead, they posit the “forward (generative) model as the core machinery of perception and action” (ibid. p. 451). Pickering and Clark call this account “integral forward modeling” (IFM). This predictive processing account holds. That a forward model drives descending predictions that “act as action commands” (Clark, 2016, p. 132). Hence, all it requires to realize a prediction is a single representation (or a descending cascade thereof) to realize both predictions of action effects and action commands.

Hence, just by the design of the predictive processing framework (and specifically IFM), the thetic perceptual representation of “awareness of prediction” is turned into a partly telic or pushmi-pullyu representation (Millikan, 1995). Simply put, predictive processing postulates a unity of action and perception: A single prediction can realize a motor command and, by the same token, the prediction of its sensory effects. Most importantly, this changes not only how predictive processing conceives of prediction. It also changes how we come to understand SoA as resulting from the prediction of action-effects, as I will discuss in the following section.

### 4.2 Predictive processing and telic action-prediction

As stated above, predictive processing holds that predictions that result from forward modeling act as both motor command *and* prediction. For SoA this means that it is not simply based on predicted action-outcomes, but also on the respective motor command (as prediction and motor command is realized by a single representation). This markedly telic notion is consistent with a non-observational, efferent conception of SoA. For agency is felt as an immediate experience tied to the very process of generating and refining predictions *qua* motor commands. Hence, it differs decidedly from a perceptual representation of agency *after the fact*, and implies that SoA is closely tied to the action itself.

This is critical to my argument, as the rejection of a holistic comparator model and specifically its application to SoA in thought is mortgaged on the notion of a perceptual and thetic representation of agency. Henceforth, with this foundation in place, we can now return to the root of our discussion: can the seminal arguments against a unified comparator model be addressed if we adopt an action-oriented predictive processing, or IFM account of SoA?

## 5 Revisiting arguments against a unified comparator model

The key arguments against a comparator model of SoA in thought were put forth by (Gallagher, 2000, 2004b, 2005; Vosgerau and Newen, 2007; Synofzik et al., 2008). These critics do not reject the comparator model as such. They accept it as a framework for SoA in bodily action. In what follows I will revisit said arguments one by one, showing that they do not hold if SoA is explained within the framework of predictive processing for both thought and bodily movement. I will start by presenting the argument against a unified account and then present the predictive processing and specifically the IFM account as a response thus debunking the argument.

## 6 Predicting thought: the infinite regress argument

The first argument addresses intentions-to-think, but it also extends to the role of prediction in thought more generally. It is premised on the notion that the original comparator model assumes that SoA results from disrupted forward modeling (Frith, 1992). When a motor command is issued, a copy of that command (called efference-copy) is created. This copy is then used to create a forward model of predicted action effects. This prediction is then compared with the outgoing motor command (notably, prior to the occurrence of sensory feedback). Frith considered this comparator mechanism as a means to monitor *intentions* (to move or think). A breakdown of the mechanism will thus result in a breakdown of self-monitoring, or “source-monitoring.”

However, such a model cannot explain SoA over thought, Gallagher argued (2004). For an intention to think that *p* is a lot like the intended thought that *p.* The notion of intention-to-think will result in a regress of intentions to think. As discussed in the previous sections, the original comparator model (Frith, 1992) has evolved since then—and as a result, SoA was not identified as an awareness of *intention* but the awareness of *prediction* (Blakemore et al., 2000, 2002; Frith et al., 2000; Jeannerod, 2009). Gallaghers argument, however, still holds true. The prediction of thought is as regressive a notion as the intention-to-think. To predict that I will think that *p* is to already entertain the thought that *p.*

To address this problem, Campbell (1999) suggested that predicting the course of thought (via efference copy) might be an unconscious process to keep thoughts on a semantic track and, therefore, not lead to an infinite regress or a double awareness of the prediction. However, (Gallagher, 2004b; Synofzik et al., 2008) argued that a non-conscious monitoring mechanism for keeping thoughts on a semantic track seems redundant. Our conscious monitoring can be considered sufficient for keeping thought on track.

### 6.1 IFM perspective

At first glance, predictive processing, and specifically IFM models of SoA over thought, face the same objection as the orthodox comparator model. Just like the orthodox account of SoA, IFM emphasizes the role of prediction: it proposes that SoA is understood as a dynamic, temporally extended process of prediction-error cancellation. Thus, SoA occurs only when the thought trajectory is successfully predicted (or enacted). And since it is precisely the conscious *prediction* of thought that is subject to infinite regress, the IFM account of SoA must also answer the regress charge.

However, first, according to IFM, the difference between (i) prediction and (ii) predicted thought (content) does not hold to begin with. Predictions are considered *telic* realizers of both motor and cognitive commands. They consist of pushmi-pullyu representations, realizing the efferent “motoric” component of thinking. Hence, to consciously predict the course of thought *is* to think that thought. This case implies neither an infinite regress nor a double awareness of thought, since there is no difference between predicting thought and thinking it as predicted.

Moreover, the infinite regress argument assumes what Lukitsch called a “static” account of SoA (2020). The static account assumes that predicting that I will think *p* is surely no different from already thinking *p*. However, first, the phenomenology of anticipation in thought is mischaracterized this way. Husserl (2013) described the phenomenology of anticipation in thought in terms of what he called “protention,” a pre-reflective, not yet explicit sense of where a thought is headed. Accordingly, when considering the flow of thought, the conscious anticipation of where thoughts are moving is embedded in a temporal shift from vague to concrete or implicit to explicit. Furthermore, Gallagher (2000) argued that the breakdown of protention is what causes passivity experiences in schizophrenia such as thought insertion and delusions of control. He states that “[w]ithout protention, thought continues, but it appears already made, not generated in my own stream of consciousness” (Gallagher, 2000, p. 225). Yet, as noted above, Gallagher holds that the breakdown of protention cannot be explained by a disruption of forward modeling. For his argument is, premised on the traditional notion of auxiliary forward modeling and thus a thetic notion of SoA.

In contrast, an IFM account of SoA, is a fitting candidate to explain the phenomenology of protention. According to such an account, the anticipation of a thought trajectory is not about predicting the course of one’s thought as *accurately* as possible. What matters is precisely the temporally extensive process of what Clark (2016) calls “sharpening” of predictions. Due to increasing the weight (and thus reducing the noise) of the error signal driven by a prediction, a prediction becomes sharper or more precise. In less technical terms this means that (i) a vague and open prediction P1 is being transformed into (ii) a specific and narrower prediction P2. In that context, however, P1 is not a prediction of P2, but rather the transformation of P1 into P2.

Finally, IFM can also account for the differences in how bodily movement and thoughts are predicted. First, while bodily movement allows for a rather precise and accurate prediction of sensory and kinesthetic feedback, the prediction of thought is considerably less precise, featuring a steep sharpening-trajectory. Most importantly, SoA, can be given the same explanation in thought and bodily action: Just like in bodily movement, SoA results from the process of continually updating predictions of the course of thinking, thus sharpening and transforming the content of thought. That said, while the just discussed argument focuses on the awareness of prediction in thought, the next seminal argument we will revisit focuses on its failure to account for SoA in unanticipated, unbidden thought.

## 7 Unbidden thought

The traditional comparator model of SoA fails to explain the difference between unbidden and inserted thought. That is to say, some of our thoughts, so-called unbidden thoughts, seem to come out of nowhere (Frankfurt, 1977). Even though we fail to predict them, we at least experience ourselves as the owners of such thoughts. Gallagher (2004b) argued that according to the comparator model, we should expect a lack of SoA over such thoughts. This is troublesome for the comparator model as it cannot explain the difference between unbidden thought and passivity experiences related to thought, such as thought insertion. Therefore, the experience of thought insertion seems to involve a more profound disruption of self-awareness than simply a lack of successful prediction (Gallagher, 2004b; Synofzik et al., 2008).

### 7.1 IFM perspective

From the perspective of an IFM account of SoA, however, unbidden thoughts do not necessarily qualify as an instance of lacking SoA. Instead, IFM accounts claim that SoA involves both successful prediction *and* prediction-error to being with. Hence, the spontaneous emergence of an unanticipated unbidden thought will not disrupt SoA as long as the thus ensuing “prediction-error” is integrated and canceled. That is, predictive processing accounts do not consider prediction-error as disruptive for agency, as long as the error-signal does not persist over time (Fletcher and Frith, 2009). In contrast, and according to IFM, passivity experiences arise when the error signal *does* persist over time and cannot be cancelled *continuously*. According to Fletcher and Frith, this leads to a vicious circle in which non-cancelled error signals lead to failed adaptive responses, which further drive the error signal (ibid.).

## 8 The redundancy of forward modeling in thought

The next argument we will look at concludes that forward modeling serves no purpose in the thinking process. It rests on the premise that the comparator model can also be seen as serving the purpose of *verifying* agency (de Vignemont and Fourneret, 2004). One can explain the evolutionary pressure for such a proper, biological function in terms of the need to verify whether external events are indeed self-produced. However, as opposed to bodily movement, there is no reason why such a biological function should have evolved for thought. Our thoughts are, by default, self-generated (Gallagher, 2004b). Thus, there is simply no evolutionary pressure for an “internal” verification mechanism to evolve, and no plausible biological function to be selected accordingly.

If the purpose of a comparator mechanism in thought cannot be the verification of agency, it must be something else. As Campbell (1999) suggested, the role of the comparator could be keeping thoughts on a semantic track. Campbell’s idea, however, was rejected on the grounds of conscious prediction leading to an infinite regress (in thought). Moreover, even if the comparator operated non-consciously, this would render it redundant once again. After, all, thoughts can be kept on track by consciously monitoring them and thus without the need of a comparator (Gallagher, 2004b).

### 8.1 IFM perspective

How this argument can be refuted is already implicitly addressed in section 7. Both, the phenomenology of SoA and IFM suggest that prediction can play a *conscious* role in thought without eliciting an infinite regress. That is, even if the prediction of the thought trajectory is itself a conscious process, it will not yield a double awareness in the form of the prediction and the corresponding subsequent thought. This is due to the temporally extensive process of “sharpening” predictions, which transforms vague and open anticipations into more precise representations, and thereby adaptively steers the course of thought. Hence, Campbell’s notion that the comparator serves the non-redundant purpose of keeping thoughts on track (rather than verifying agency) is consistent with and delineated in more detail by an IFM account of SoA.

## 9 Hyperreflexivity

The original comparator model (Frith, 1992; Campbell, 1999) suggested that SoA involves some form of introspective awareness (Gallagher, 2004b), and that the lack of such introspective awareness arises due to a disruption of the comparator. Yet, there is evidence that passivity experiences do not simply involve a *lack* of introspection. On the contrary, patients with schizophrenic symptoms such as passivity experiences report that they have a *heightened* awareness of bodily sensations and pay increased attention to aberrant experiences (of their body and world). If anything, people with schizophrenia report that too much reflection fosters an increased presence of otherwise less salient bodily sensations (Sass, 2003b, 2003a, 2014).

However, this inconsistency regarding the disruption of introspective awareness versus an increased introspective awareness only affects the original comparator account (i.e., Campbell, 1999; Frith, 1992). More recent comparator models focus on the awareness of prediction, rather than introspection (Frith, 2012). Hence, these accounts do not contradict reports of hyperreflexivity—but they do not explain the phenomenon either. A predictive processing account of SoA provides more promising prospects, however. Even more so, it can plausibly account for *both* the disruption of hyperreflexivity and a disruption of SoA, or so I shall argue in the subsequent section.

### 9.1 Predictive processing response

An IFM model of SoA does not only sidestep the argument from hyperreflexivity presented above, but provides an elegant explanation for hyper-saliency in bodily sensation and hyperreflexivity in thought. A terminological note is in order here: Hyperreflexivity is spelled with an “x.” The literature refers to the phenomenon as *reflective* hyperreflexivity, characterizing a situation where an increased awareness of bodily sensations leads to a reflective attitude towards these sensations (Sass, 2003b). Predictive processing accounts are in a particularly good position to explain this.

Fletcher and Frith (2009) propose such an of schizophrenic passivity experiences. In general, predictive processing posits a multilevel hierarchy of prediction units (called “representation units”) and error units, respectively. When a higher-level representation unit fails to predict the state of its corresponding lower-level representation unit, an error signal is propagated backwards. However, this process is not binary, but involves degrees of confidence in both prediction and prediction error. For example, an accurate prediction may still be subject to change and refutation if a corresponding error signal is highly weighted. This way, predictions can be “sharpened” (Clark, 2016) even when they are successful. According to Fletcher and Frith, the increased salience of lower-level sensation and perception in schizophrenia might arise due to an (unaccounted) increased weighting of error signals. Such error signals should be ignored because they correspond to accurate higher-level predictions. However, since the faulty prediction error triggers an update of the higher-level prediction, a vicious increase in prediction error ensues: the newly updated prediction will not be able to account for and cancel the prediction error, since the prediction itself now becomes less accurate. This will propagate an error signal further upward in the hierarchy, inducing delusional thinking to explain (away) the unaccounted-for prediction error.

Most importantly, the theory of how SoA is disrupted by the failure of continuous prediction error minimization follows naturally from this explanation. Due to the false weighting of prediction error, the corresponding predictions cannot be updated. As a consequence, these predictions will also fail to reduce the error signal. Yet, according to the predictive processing theory of agency experience discussed above, this is a necessary condition for the emergence of SoA (Gerrans, 2014; Clark, 2016; Lukitsch, 2020). That being said, we can now turn to the final argument again a unified comparator account.

## 10 The problem of selectivity, specific content, and episodes

The final argument against a comparator model of SoA over thought concerns the selectivity of passivity experiences. A comparator model of disrupted SoA implies a general breakdown of the comparator mechanism (Synofzik et al., 2008), suggesting that all thoughts must be affected and thus lack SoA. Yet, this is not what happens. Some passivity experience, such as thought insertion (i.e., the experience that thoughts are inserted into one’s mind from the external world) are often limited to short episodes and constrained to specific thought content. Only some thoughts and only thoughts with *specific content* coincide with the symptom. For instance, an inserted thought might be attributed to a specific person, containing recurring narratives.

Such selective experiences can be characterized as a disruption of SoA over thought. Yet, the orthodox comparator model does not explain why the comparator fails selectively. This is a problem for what Pickering and Clark (2014) called auxiliary forward modeling: the traditional comparator account would have it that the comparator is a special-purpose module in a larger cognitive architecture. The question then arises as to why, if such a module is disrupted, we do not experience a general disruption of the SoA, which affects all our thoughts.

### 10.1 Predictive processing response

In contrast to auxiliary forward modeling, predictive processing proposes an integral notion of forward modeling (as discussed in section 4.1). Accordingly, the comparator mechanism is the basic processing principle of a cognitive system, rather than an isolated, compartmentalized module. Hence, an integral approach is consistent with the notion that only *specific predictions* are driven by a falsely weighted error signal (rather than the entire machinery of forward modeling). By the same token, only the thematic content of these compromised predictions might be affected, explaining why the disruption of SoA coincides with thematically particular delusion.

As described earlier in the context of hyperreflexivity, according to IFM, the increased salience of lower-level sensation and perception in schizophrenia might arise due to an (unaccounted-for) increased weighting of error signals (Fletcher and Frith, 2009). Thus, such persistent false prediction errors will propagate upwards in the processing hierarchy, eventually forcing the subject to abandon higher-level predictions, for instance, in favor of delusional beliefs. Such beliefs then, might occur at the level of hyper-priors trying to explaining the persistence of lower-level error signal. Considering this, the thematic specificity can be explained by the fact that higher-level predictions (such as delusions, thought insertion, or thought withdrawal) are driven by a specific instance or prediction error rendering bodily sensation overly salient. In this sense, IFM is a profoundly “explanationist” (Stone and Young, 1997) account, in that it identifies the thematic content of delusions as an attempted explanation of aberrant lower-level experiences.

Most importantly, this predictive processing theory of the selectivity of delusional thought content arises naturally from its explanation of disrupted SoA. For after all, persistent and misweighted prediction errors at lower levels result in “delusional” predictions at higher levels. Therefore, the temporally extensive process of prediction error cancellation necessary for SoA will not occur, explaining why only some predictions (and their thematic content) will be affected by a lack of SoA.

Taken together, the arguments just discussed could be seen as the reason for the rejection and a major barrier for a unified comparator model of SoA. Considering that these barriers are thus removed, how does a unified predictive processing theory of SoA fare against the backdrop of recent empirical evidence?

## 11 Empirical prospects of a unified comparator model

Although the primary goal of this article is to establish the conceptual possibility of a unified predictive processing account of the experience of agency, we will consider whether such a defense was worthwhile at all in light of recent findings in the study of SoA. First of all, neurocognitive models correlate the neural constituents of SoA with activity in specific brain areas, such as the anterior insula, the angular gyrus, and attenuated activity in the right inferior parietal cortex (Spence et al., 1997; Blakemore et al., 2000; Frith et al., 2000; Haggard, 2017). Yet, these studies provide no evidence for a unified machinery of SoA for bodily action and thought. To my knowledge, no studies have focused on the *implicit* SoA (rather than agency judgments) over thought and action and succeeded in identifying their common neural basis. Considering this, an IFM account of SoA asserts, first and foremost, that the functional, conceptual basis for SoA is homologous across action and thought. It does not, therefore, require that there be a common neural basis. Hence, any finding that there is, or is no common neural correlate for SoA across thought and bodily action is of limited relevance at least to my argument. That is, a neurobiologically distinct basis for SoA in thought and bodily action does not count as evidence against a unified account if it does not entail a conceptual of functional difference.

There is evidence, however, that SoA can be homologous in mental and bodily action. This is indicated by finding that mental action is accompanied by intentional binding (Lopez-Sola et al., 2021), suggesting that “mental actions and motor actions produce a comparable sense of agency at a conceptual level” (ibid. p. 13). More importantly, there is evidence suggesting some unified machinery for the feeling of *effort* (Bermúdez, 2024; Bermúdez and Massin, 2023). This finding is of particular interest for a unified predictive processing account of SoA as the necessary presence of prediction error in experiences of agency is reflected in a feeling of effort (Gerrans, 2014; Lukitsch, 2020).

Even though the focus of neurocognitive models of SoA is on bodily actions, it is still worth discussing the extent to which this model is related to the latest findings on the multidimensional embedding of SoA in the neural machinary of self-awareness. That is to say, the predictive processing theory I draw on in this article has an overly limited focus on SoA as resulting from prediction error minimization over time. Yet, SoA does not occur in isolation (cf. Synofzik et al., 2008).

### 11.1 SoA and the multidimensional sense of self

More recently, Di Plinio and colleagues suggested that SoA is embedded in a broader system of self-processing mechanisms involving both intrinsic and extrinsic self-processing (Di Plinio et al., 2020b). In this context, intrinsic self-processing consists of assigning sensory input to the self as personally relevant, thus drawing on information from memory and self-narrative. It is correlated with activity in sensory and default mode networks. Extrinsic self-processing, on the other hand, includes the self-relatedness of action-consequences and thus SoA proper. It is correlated with activity in “intermodular connections of a frontoparietal module including the premotor cortex, supramarginal gyrus, and dorsal precuneus” (Di Plinio et al., 2020c, p. 1764). This intermodular system is specifically associated with individual differences in prospective intentional binding (ibid.). Moreover, it has been suggested that both intrinsic and extrinsic self-processing occur during the performance of voluntary actions (Di Plinio et al., 2020b). While these processes are functionally separate circuitry, they interact to give rise to a multidimensional self in which SoA is only a (possibly insufficient) component.

Furthermore, SoA is not a stable phenomenon, modulated solely by the sensorimotor trajectory of a given situation. Rather, SoA is subject to “implicit learning,” in that it “can be retrospectively influenced by the nature of the ongoing event, suggesting that predictive mechanisms rely on multiple temporal scales” (Di Plinio et al., 2020a, p. 10). This shows that SoA is might not only be constituted by the adaptive nature of an on-going action itself, but by the retrospective, adaptive re-evaluation of SoA over time.

Finally, while SoA is a non-stable phenomenon embedded in a wider framework of self-processing, it is even affected by personality traits and contextual beliefs (Obhi et al., 2012; Barlas and Obhi, 2014; Hascalovitz and Obhi, 2015; Di Plinio et al., 2019). Hence, taken together, these recent findings raise the question of whether SoA can be explained in isolation in terms of integral forward modeling and prediction error minimization.

### 11.2 Embedding of SoA across the predictive processing hierarchy

While these findings show that SoA cannot be treated in isolation of other predictive mechanisms in which it is embedded, they also suggest that the predictive processing framework is conceptually well suited to treat SoA as an integrated, dynamic phenomenon. Di Plinio et al. acknowledge this suggesting “that the relationship between the SoA and global modularity might be explained by the reliance of prospective intentional binding effects on multimodal predictive mechanisms constituting a general principle of brain functioning” (Di Plinio et al., 2020c, p. 1776). That is to say, it is intrinsic to the functional architecture of predictive processing that the brain is said to continually compute and adapt the confidence in its own predictions. It does so across several layers of its predictive processing hierarchy, thus explaining why contextual and past information can change the experience of agency in various ways.

Moverover, higher-level priors such as beliefs can be understood as drivers of prediction error in lower-level systems (such as perceptual and sensorimotor systems) since systematically inaccurate beliefs cannot “explain away” the corresponding error signal (Fletcher and Frith, 2009). Note that such higher-level priors could involve personality and basic forms of self-narrative and hyperpriors about self-hood. Most notably, this makes predictive processing as a particularly compelling account to explain both the influence of higher-level predictions on SoA and the disruption of SoA by inaccurate hyperpriors.

With this in mind, further research could establish whether SoA is disrupted primarily due to top-down influence of higher-level priors and hyper-priors or rather by lower-level predictions. Especially the former (i.e., higher-level priors) are disrupted in *positive* symptoms of schizophrenia, as patients entertain false beliefs about the internal nature of thought and their bodily agency. On the other hand, phenomenological approaches in psychopathology have suggested that aberrant self-experience in schizophrenia first manifests in the form of hyper-salient bodily sensations and a heightened awareness of otherwise attenuated bodily experiences (Parnas and Sass, 2011). This, in turn, can be explained by the presence of misweighted lower-level prediction error, subsequently driving inaccurate higher-level beliefs.

Most importantly, and considering the goal of this article, it will be essential to investigate whether the inability of higher-level predictions to cancel prediction error will be sufficient to disrupt SoA over thoughts. As of now, SoA is seldom studied as an *implicit* experience or feeling *in mental action* or thought, but rather as explicit judgement or understood in the context of metacognitive monitoring (e.g., Carruthers, 2012). One possibility lies in the finding that mental action is accompanied by intentional binding (Lopez-Sola et al., 2021), providing a paradigm to further investigate a possibly shared processing principle for SoA. By identifiying a shared functional basis for both SoA in thought and action one could then proceed to further investigate shared neural underpinnings for such functionally homologous experiences. Yet, as discussed above, while this will provide another argument for a unified comparator model, shared neural underpinnings are not a necessary condition for a unified account to begin with.

## 12 Conclusion

I argued that a comparator model of SoA is conceptually possible. To show this, I portrayed a refined, partly telic notion of agency experience as the temporally extensive process of prediction error cancellation, based on the principles of predictive processing and integral forward modeling. This theory of SoA does not face the arguments against the original unified comparator model. It is *conceptually* possible. Yet, showing that a unified model is conceptually possible is not an empirical finding. It is beyond the scope of this article to present such an empirical account. Yet, the initial rejection of a unified comparator model was not a rejection on grounds of inconsistencies in neuroscientific evidence but due to conceptual inconsistencies. It was largely based on the notion that prediction and descriptive, thetic representation cannot play the same functional, causal role in thought and bodily action. My goal was to show that prediction, as conceived by predictive processing and IFM, can assume such an integral role, thus paving the way for a unified comparator account of agency experience.

## Data Availability

The original contributions presented in the study are included in the article/supplementary material, further inquiries can be directed to the corresponding author.

## Footnotes

^1^It must be added that such more recent versions of the comparator model distinguish between the experience of agency as (i) resulting from judgements of agency (as originally suggested by Wegner, 2002; Wegner and Wheatley, 1999) and (ii) the first-hand phenomenal feeling of controlling an action (as in Haggard, 2003, 2017). Bayne and Parcherie call this composition an interplay of “narrators and comparators” (Bayne and Pacherie, 2007). The role of the comparator, according to this framework, is the realization of the first-hand phenomenology of agency. The *posthoc* narrative construction of agency also involves prediction and comparison but, in contrast, does so only on the level of rational control of action (Pacherie, 2008).
